# Empyema caused by *Streptococcus constellatus*: a case report and literature review

**DOI:** 10.1186/s12879-021-06955-2

**Published:** 2021-12-20

**Authors:** Jingyan Xia, Lexin Xia, Hui Zhou, Xiuhui Lin, Feng Xu

**Affiliations:** 1 Department of Radiation Oncology, The Second Affiliated Hospital, Zhejiang University School of Medicine, Hangzhou, 310009 China; 2 Department of Infectious Diseases, The Second Affiliated Hospital, Zhejiang University School of Medicine, Hangzhou, 310009 China

**Keywords:** *Streptococcus constellatus*, Empyema, Next-generation sequencing

## Abstract

**Background:**

*Streptococcus constellatus* is a member of *Streptococcus anginosus* group (SAG) that tends to cause pyogenic infections in various sites. However, *Streptococcus constellatus* is easily ignored by routine clinical laboratory tests for its prolonged anaerobic culture environment.

**Case presentation:**

A 71-year-old man was admitted to our hospital due to productive cough, fever, chest pain and shortness of breath for 3 weeks. Chest computed tomography showed patchy opacities and right-sided pleural effusion, so a chest tube was inserted and purulent and hemorrhagic fluid was aspirated. The routine etiological examinations of the pleural effusion were all negative, and next-generation sequencing (NGS) detected *Streptococcus constellatus*. Intravenous piperacillin-tazobactam 4.5 g every 8 h was used accordingly. The patient recovered and subsequent chest computed tomography confirmed the improvement.

**Conclusions:**

We reported a case of empyema secondary to *Streptococcus constellatus* infection, which was identified by NGS, instead of bacterial culture. This case highlights the utility of NGS in detecting pathogens negative in traditional bacterial tests.

## Background

Empyema is a common complication of lung bacterial infections, but it is rarely reported to be caused by *Streptococcus constellatus* (*S. constellatus*). *S. constellatus* is one of the *Streptococcus anginosus* group (SAG), a subgroup of viridans streptococci, widely distributed in the oral cavity, nasopharynx, gastrointestinal tract, and vagina in 15–30% of healthy people [1]. SAG tends to cause pyogenic infections, such as dental, peritonsillar and sinus abscesses [2].

As a member of SAG, *S. constellatus* induces abscesses mainly in respiratory tract, brain, liver, bone and soft tissues [3–5]. SAG infection has a strong male predominance [6, 7]. The symptoms of pulmonary infection caused by *S. constellatus* include shortness of breath, chest pain, cough, and fever [6]. Laboratory tests often suggest leucocytosis, neutrophilia, abnormal liver function, and hypo-albuminaemia [6]. The median stay in hospital was 34 days [6].

Here, we reported the use of next-generation sequencing (NGS) in detecting *S. constellatus* as the pathogen for a case of empyema of unknown causes. This case highlights the use of NGS in etiology diagnosis and guiding the treatment in empyema.

## Case presentation

A 71-year-old male was admitted to the hospital due to productive cough along with low grade fever, chest pain and shortness of breath. His past medical history included hypertension and glaucoma, and he took irbesartan regularly (150 mg per day). The patient did not smoke cigarettes, drink alcohol or use recreational drugs. No relevant travel history or contact history were detected. The patient had no food or drug allergies.

Three weeks before admission, the patient began to have productive cough, with chest tightness and a temperature of 38 °C. After 2 weeks of progressive symptoms, the patient visited the local hospital. He reported pleuritic chest pain of visual analogue scale score 2. His vital signs and other physical examination results were reported as normal. Initial blood test showed elevated white blood cell (WBC) count (14.8 **×** 10^9^/L) and C-reactive protein (CRP) level (86 mg/L) as well as liver enzyme elevation. Other laboratory test results were normal. Chest computed tomography (CT) revealed patchy opacities in both lower lobes and a small amount of right-sided pleural effusion. He was then admitted to the local hospital and received intravenous sulperazon (cefperazone–sulbactam) 2.0 g once every 8 h, but symptomatic improvement was not noted. Repeated chest CT scan revealed increased pleural effusion in the right. Subsequently, the patient was transferred to our hospital for treatment.

**Fig. 1:**
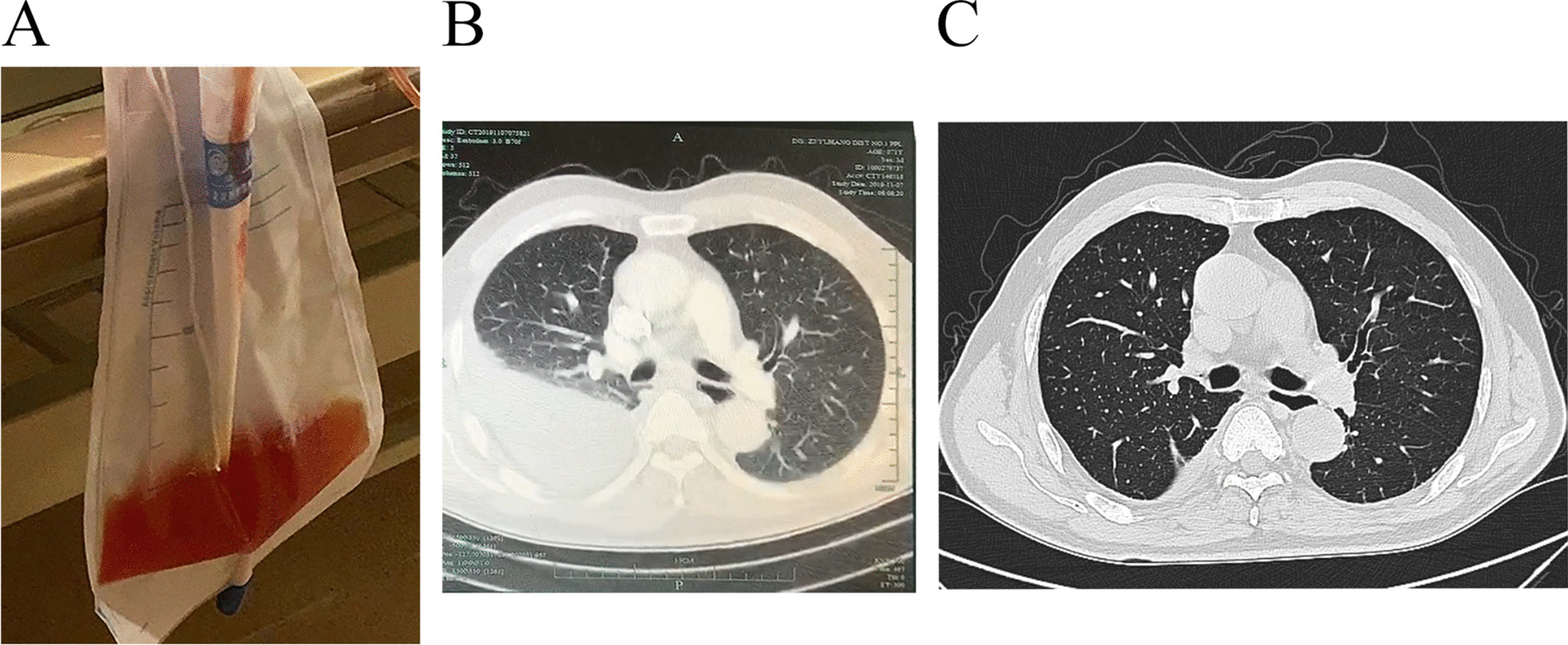
The appearance and CT scan of pleural effusion. **A** Appearance of the pleural effusion. **B** Chest CT showed moderate, right-sided pleural effusion on the day of admission. **C** Chest CT showed significantly reduced, small amount of right-sided pleural effusion 7 days after the admission.

On the admission, his temperature was 37.8 °C, pulse rate 109 beats/min, respiratory rate 18 breaths/min, blood pressure 145/87 mmHg, and oxygen saturation 98% on room air. The patient reported no night sweats, weight loss, joint pains, or myalgias. Pulmonary auscultation found decreased breath sounds on both lower fields. No icterus or lymphadenopathy was detected. Thoracocentesis was performed immediately and a chest tube was introduced. Purulent and hemorrhagic fluid was aspirated (Fig. 1A), and laboratory tests of the pleural effusion revealed an exudate (fluid protein 25.70 g/L, serum protein 28.70 g/L [normal: 35.0–55.0 g/L], fluid lactate dehydrogenase 621 U/L, serum lactate dehydrogenase 355 U/L [normal < 243 U/L]). Other pleural fluid analysis showed: WBC 5000/µL with 88% neutrophils, red blood cells 21,000/µL, fluid glucose 7.31 mmol/L, and fluid adenosine deaminase 18 U/L. Pleural fluid smear, gram stain, bacterial culture, acid-fast bacilli culture and smear, and cytology were all negative. Blood culture was also negative. He was diagnosed as empyema and intravenous tazocin (piperacillin-tazobactam) 4.5 g once every 8 h was initiated.

During the treatment, repeated blood and fluid cultures were all negative, so the pleural effusion was sent for NGS. Deoxyribonucleic acid (DNA) was extracted directly from the pleural fluid. The extracted DNA was amplified, purified, and sonicated to a size of 200–300 bp. Fragmented DNA was end repaired to construct DNA libraries, and supplemented with adapter overnight followed by polymerase chain reaction (PCR) amplification. Qualified DNA libraries were sequenced using the BGISEQ-100 platform. We generated high-quality sequencing data by removing low-quality reads, low-complexity reads, and reads shorter than 35 bp. Human data were mapped to a human reference (hg19) and excluded. The remaining sequencing data were aligned to the bacterial, virus, and fungal databases. All pathogen sequence reference databases were from National Center for Biotechnology Information (NCBI) (ftp://ftp.ncbi.nlm.nih.gov/genomes/).

NGS identified 14 out of 19317 reads uniquely corresponding to *S. constellatus*. Although the number of the reads was small, *S. constellatus* was the only pathogen detected. Tazocin was used continuously for 12 days, then followed by moxifoxacin 400 mg per day for a total of 14 days. Meanwhile, the pleural effusion was drained continuously through the chest tube. The patient claimed gradually resolved symptoms under antibiotic treatment and effusion drainage, and subsequent CT scanning confirmed the improvement (Fig. 1B, C).

## Discussion and conclusions

In this case, we reported a case of empyema caused by *S. constellatus*. The identification of *S. constellatus* is sometimes difficult because of the prolonged anaerobic culture environment [8]. During the recent decades, faster and more convenient technical methods have been applied to investigate the pathogens of infectious diseases. For example, real-time PCR assays targeting the cpn60 and 16S ribosomal ribonucleic acid (rRNA) genes were used to detect SAG [1, 8]. Most of the *S. constellatus* (96%), *S. intermedius* (94%), and *S. anginosus* (60%) strains were correctly identified by targeting cpn60 [1]. 16S ribosomal deoxyribonucleic acid (rDNA) sequencing of purulent fluid obtained from a muscle abscess aspirate was successfully used to diagnose pyomyositis caused by SAG [8]. Rapid diagnostic kits [9] and matrix-assisted laser desorption/ionization time-of-flight mass spectrometry (MALDI-TOF MS) [10] were also used in clinical laboratories to identify SAG. In recent years, the application of NGS shed light on detecting pathogens more efficiently. NGS can sequence thousands to billions of DNA fragments simultaneously, making quick identification of the pathogens in culture-negative specimens possible [11]. Compared with 16S rRNA sequencing, which identifies one organism at a time, NGS can provide information on multiple organisms simultaneously [12]. Although NGS has its own drawbacks such as shorter read length and decreased raw accuracy in copy number variants (CNVs) or other structural variants, the development of culture independent NGS technology still offers unbiased, rapid etiology diagnosis for the entire microbial community [13].

In this case, *S. constellatus* was detected by NGS rather than traditional bacterial culture, and various reasons could contribute to this matter. Frist, *S. constellatus* is a facultative anaerobic organism sometimes difficult to culture, especially when not specifically requested. Secondly, the patient started with antibiotics before his admission to our hospital. It may reduce culture-dependent bacterial detection. Compared to the difficulty of traditional culture, NGS was able to amplify and detect cell-free DNA and DNA fragments in dead cells, making etiology diagnosis possible. NGS should be considered in the following occasions: pathogens not detected in clinical specimens; patient already receives antibiotics; and suspicious of uncommon pathogens.

According to the literatures, among a collection of 423 clinical SAG, 1.4% of the strains were of intermediate susceptibility to penicillin and none exhibited high-level resistance to gentamicin. All the strains were susceptible to cefotaxime, vancomycin and teicoplanin [14]. Clindamycin, doxycycline, amoxicillin, and metronidazole could be used for *S. constellatus* infection [15, 16]. Although *S. constellatus* is not a commonly detected pathogen causing pleural effusion, we still need to pay attention to its pathogenicity in clinical practice.

Collectively, this case reported an empyema secondary to *S. constellatus* infection, where NGS helped to determine the pathogen. NGS is of great significance to infection of unknown pathogens, and we expect further application of advanced sequencing technologies in infectious diseases in the future.

## Data Availability

All data generated or analyzed during this study are included in this published article. All data and materials are available with the corresponding author.

## Abbreviations

SAG: *Streptococcus anginosus* group
NGS: Next-generation sequencing
WBC: White blood cell
CRP: C-reactive protein
CT: Computed tomography
rRNA: Ribosomal ribonucleic acid
DNA: Deoxyribonucleic acid
PCR: Polymerase chain reaction
NCBI: National Center for Biotechnology Information
rDNA: Ribosomal deoxyribonucleic acid
MALDI-TOF MS: Matrix-assisted laser desorption/ionization time-of-flight mass spectrometry
CNV: Copy number variant
